# Supplementary material for: The impact of cytochrome P450 3A genetic polymorphisms on tacrolimus pharmacokinetics in ulcerative colitis patients
Source: PLoS One. 2021 Apr 22;16(4):e0250597. doi: 10.1371/journal.pone.0250597 (PMC8062093; doi:10.1371/journal.pone.0250597)
Supplement: S1 Table — (PDF) [file pone.0250597.s001.pdf]

Table S1. Combination of CYP3A4 and CYP3A5 gene polymorphisms

	*CYP3A4*1/*1*		*CYP3A4*1/*1G+ *1G/*1G*		
	*CYP3A5*1/*1+*1/*3*	*CYP3A5 *3/*3*	*CYP3A5*1/*1+*1/*3*	*CYP3A5 *3/*3*	P-value
Number of cases	0	30	13	2	
C/D ratio when reached high trough†			74.9 ± 32.6	173.2 ± 19.0	0.001##
C/D ratio after 2-5 days†			51.7 ± 23.0	173.2 ± 19.0	< 0.0001###
C/D ratio after 7 days†			72.0 ± 44.2	293.1 ± 68.5	< 0.0001###
C/D ratio after 14 days†			65.6 ± 26.6	172.4 ± 22.8	< 0.001###

# = P < 0.05, ## = P < 0.01, ### = P < 0.001

†mean ± SD, ng/mL per mg/kg

C/D ratio: Tac concentration and dose ratio; CYP3A4: cytochrome P450 family 2 subfamily A member 4; CYP3A5: cytochrome P450 family 2 subfamily A member 5; SD: standard deviation
