# Supplementary material for: The impact of cytochrome P450 3A genetic polymorphisms on tacrolimus pharmacokinetics in ulcerative colitis patients
Source: PLoS One. 2021 Apr 22;16(4):e0250597. doi: 10.1371/journal.pone.0250597 (PMC8062093; doi:10.1371/journal.pone.0250597)
Supplement: S2 Table — (PDF) [file pone.0250597.s002.pdf]

Table S2. Adverse events summary

Adverse event	Number (%)
Renal impairment	14 (31)
Hypomagnesemia	12 (26)
Hyperkalemia	6 (13)
Neuropathy	5 (11)
Nausea	5 (11)
Headache	4 (9)
Tremor	3 (7)
Hepatopathy	2 (4)
Hyponatremia	1 (2)
Fatigue	1 (2)
